# Correction of Chloride Transport and Mislocalization of CFTR Protein by Vardenafil in the Gastrointestinal Tract of Cystic Fibrosis Mice

**DOI:** 10.1371/journal.pone.0077314

**Published:** 2013-10-24

**Authors:** Barbara Dhooghe, Sabrina Noël, Caroline Bouzin, Gaëtane Behets-Wydemans, Teresinha Leal

**Affiliations:** 1 Louvain centre for Toxicology and Applied Pharmacology (LTAP), Institute of Experimental and Clinical Research (IREC), Université catholique de Louvain, Brussels, Belgium; 2 IREC Imaging Platform/Pole of Pharmacology and Therapeutics, Institute of Experimental and Clinical Research (IREC), Université catholique de Louvain, Brussels, Belgium; Inserm, France

## Abstract

Although lung disease is the major cause of mortality in cystic fibrosis (CF), gastrointestinal (GI) manifestations are the first hallmarks in 15–20% of affected newborns presenting with meconium ileus, and remain major causes of morbidity throughout life. We have previously shown that cGMP-dependent phosphodiesterase type 5 (PDE5) inhibitors rescue defective CF Transmembrane conductance Regulator (CFTR)-dependent chloride transport across the mouse CF nasal mucosa. Using F508del-CF mice, we examined the transrectal potential difference 1 hour after intraperitoneal injection of the PDE5 inhibitor vardenafil or saline to assess the amiloride-sensitive sodium transport and the chloride gradient and forskolin-dependent chloride transport across the GI tract. In the same conditions, we performed immunohistostaining studies in distal colon to investigate CFTR expression and localization. F508del-CF mice displayed increased sodium transport and reduced chloride transport compared to their wild-type littermates. Vardenafil, applied at a human therapeutic dose (0.14 mg/kg) used to treat erectile dysfunction, increased chloride transport in F508del-CF mice. No effect on sodium transport was detected. In crypt colonocytes of wild-type mice, the immunofluorescence CFTR signal was mostly detected in the apical cell compartment. In F508del-CF mice, a 25% reduced signal was observed, located mostly in the subapical region. Vardenafil increased the peak of intensity of the fluorescence CFTR signal in F508del-CF mice and displaced it towards the apical cell compartment. Our findings point out the intestinal mucosa as a valuable tissue to study CFTR transport function and localization and to evaluate efficacy of therapeutic strategies in CF. From our data we conclude that vardenafil mediates potentiation of the CFTR chloride channel and corrects mislocalization of the mutant protein. The study provides compelling support for targeting the cGMP signaling pathway in CF pharmacotherapy.

## Introduction

Cystic fibrosis (CF) is a life-shortening genetically inherited disease caused by mutations that alter the expression and/or the activity of the CF Transmembrane conductance Regulator (CFTR) protein. CFTR functions as a transepithelial low-conductance chloride channel [1], [2] and as a regulator of other membrane transporters, most notably of the epithelial sodium channel ENaC, upregulated in CF [3], [4]. The most prevalent F508del-CFTR mutation, present in ∼70% of CF chromosomes, and in ∼90% on at least one allele, of CF patients [5], corresponds to deletion of the phenylalanine 508 in the 1480 polypeptide chain. It causes defective folding of the protein that is mostly retained in the endoplasmic reticulum (ER), is tagged for premature degradation by the ubiquitin-proteasomal pathway and is only marginally expressed at the surface of epithelial cells [6]. Most of the emerging therapies have focused on correcting the trafficking defect in order to rescue the mutant protein to the cell surface [7]–[11]. However, the rescued misfolded F508del protein displays altered gating properties with reduced chloride channel opening [12] and accelerated endocytosis and recycling [13]–[16] with reduced residence time in the apical membrane. A recent study [17] has identified hepatocyte growth factor, a compound under clinical trial for different conditions such as myocardial infarction and acute hepatic failure, as an agent able to increase the residence time of F508del-CFTR in the cell membrane. Studies on modulation of endocytic activity of misfolded integral proteins with formation of intracellular aggregates and autophagy as well as the consequences of these deregulated processes in CF disease are under investigation [18]. Pharmacological chaperones interacting with F508del-CFTR itself, facilitating its folding and cellular processes and agents regulating proteostasis by modulating the cellular quality-control machinery or inhibiting proteasome activity may have therapeutic potential for CF. Such agents have been termed “correctors” [5], [7]–[11], [17], [19]–[21] even if proteasome inhibitors do not adequately rescue F508del-CFTR [22]. Agents increasing the PKA-regulated open probability of the protein channel expressed at the plasma membrane have been termed “potentiators” [8], [20], [21], [23]. Ivacaftor, the only approved CFTR potentiator, increases the channel activity with documented clinical improvements [23]. Correctors under investigation, such as lumacaftor and miglustat, have, at the best, modest beneficial clinical effects [7], [11]. Targeting the multiple molecular defects caused by the F508del mutation may require a therapy combining correctors and potentiators or the use of a single therapeutic agent with both correcting and potentiating properties [19], [20], [21].

CF epithelia are characterized by defective transepithelial ion transport, namely reduced chloride transport and increased sodium transport, which has long been assessed by measuring nasal potential difference (PD) [24], [25]. More recently, the nasal PD test has proven helpful for assisting in the efficacy of basic CFTR therapeutics [7], [11], [26]. Despite the clear link between abnormal ion transport and CF, the pathogenesis of the disease is complex and is still a subject of debate. It involves multiple organs, including airways, pancreas, intestine, liver, sweat glands and vas deferens, but lung and digestive disease are the major causes of morbi-mortality. Respiratory disease is characterized by progressive sino-pulmonary disease that develops largely as a consequence of the abnormal ion transport and the inability to efficiently hydrate the epithelial surface liquid layer [27]. The resulting dehydrated mucus compromises mucociliary clearance and makes CF airways vulnerable to chronic neutrophil-dominated inflammation and infection finally leading to respiratory failure. Digestive disease with pancreatic exocrine insufficiency is seen in 85–90% of patients with CF. Although lung disease is the major cause of mortality, gastrointestinal (GI) disease is the first hallmark of CF in a significant number (15–20%) of affected newborns that present with obstructive meconium ileus, and remains a major cause of morbidity throughout life. Status of GI expression serves as a marker of disease severity. As in airways, mucus secretions in the GI tract are more viscous and dehydrated, also as a result of abnormal fluid flow [28].

It is generally agreed that, in intact cells, cAMP- and protein kinase A-dependent phosphorylation is the major mechanism regulating CFTR activity [29]. It has also been recognized that cGMP-dependent protein kinase G signals CFTR channel gating activity [30] and regulates intestinal fluid and ion homeostasis [31]. However, the mechanisms underlying modulation of CFTR activity by intracellular accumulation of cGMP are still being sought. This can be achieved by stimulating its formation (*i.e*. by means of guanylyl cyclase agonists) or by inhibiting its degradation (*i.e*. by means of phosphodiesterase (PDE) inhibitors). Vardenafil, sildenafil and taladafil are highly selective inhibitors of cGMP-dependent PDE type 5 commonly used for improving erectile dysfunction [32]. In the context of CF, it has been shown that treatment with sildenafil, applied at doses ∼1 000 times larger than those used for erectile dysfunction, is able to correct the mislocalization and defective anion transport function of the F508del-CFTR protein in nasal epithelial cells harvested from CF patients [33]. We have shown that intraperitoneal injection [34] or inhalation [35] of therapeutic doses of PDE5 inhibitors to F508del-CF mice rescue CFTR-dependent chloride transport across the nasal mucosa. We hypothesized that *in vivo* treatment with a clinical dose of vardenafil corrects F508del-CFTR chloride channel dysfunction and mislocalization in another CF target tissue. Vardenafil was selected as a representative PDE5 inhibitor for its longer-lasting and more potent CFTR activating effect and its larger water solubility than those of sildenafil [34], [35]. CFTR function was studied in the rectal mucosa, representative of the GI tract, by measuring *in vivo* transrectal PD in a clinically relevant F508del-CF mouse model [36]. The effect of vardenafil on mislocalization of F508del-CFTR protein was assessed in distal colon pieces excised from mice.

## Results

Both male and female mice have been used in the study. As no gender-related difference was observed, data from both genders have been pooled.

### Baseline and Stimulated Transrectal PD Values in Nontreated F508del-CF and Wild-type Mice

Before testing whether vardenafil can rescue CFTR-mediated chloride transport across the GI epithelia, we first determined *in vivo* ion transport properties of the rectal mucosa in CF mice homozygous for the F508del mutation built in the 129/FVB background [36] and in their normal homozygous littermates. Similar to the nasal mucosa of CF patients [7], [11], [24]–[26], [37] and mice [34], [35], [37], [38], the rectal mucosa of F508del-CF mice displays typical CF ion transport abnormalities. Transrectal PD recording started only when a stable value had been obtained. As illustrated in representative tracings (Figure 1), in comparison with wild-type, F508del-CF mice showed 1) basal hyperpolarization (stable voltage value more electrically negative); 2) increased response after perfusing the rectal mucosa with a buffered Ringer solution containing amiloride (to inhibit ENaC activity) and barium (to block potassium channels); 3) reduced repolarization after perfusing the mucosa with an amiloride- and barium-containing chloride-free solution of sodium gluconate to induce CFTR-mediated chloride flux; and 4) reduced repolarization after addition of forskolin, an adenylate cyclase agonist, to the chloride-free solution in order to maximally stimulate cAMP-dependent CFTR-mediated chloride transport. Mean values of transrectal PD are illustrated in Figure 2. Mean absolute basal values in F508del-CF mice were roughly twice as large as in wild-type mice. In both groups, the values nearly fell to zero under the effect of amiloride, the changes amounting to 40.2±4.0 mV in F508del-CF mice *vs* 20.0±1.8 mV in wild-type mice (mean ± SEM; p<0.001). Chloride transport was evaluated by the difference between the PD value measured at the end of perfusion with zero-chloride solution containing forskolin and that measured at the end of perfusion with Ringer solution containing amiloride and barium; it was reduced by half in F508del-CF mice (−4.2±0.5 mV) compared to that measured in wild-type mice (−9.4±0.9 mV; p = 0.002), integrity of chloride transport being characterized by a more marked repolarization, *i.e.* more negative PD values. These data indicate that the rectal mucosa of F508del-CF mice reproduces nasal transepithelial ion transport abnormalities, the hallmarks of CF disease.

**Figure 1:**
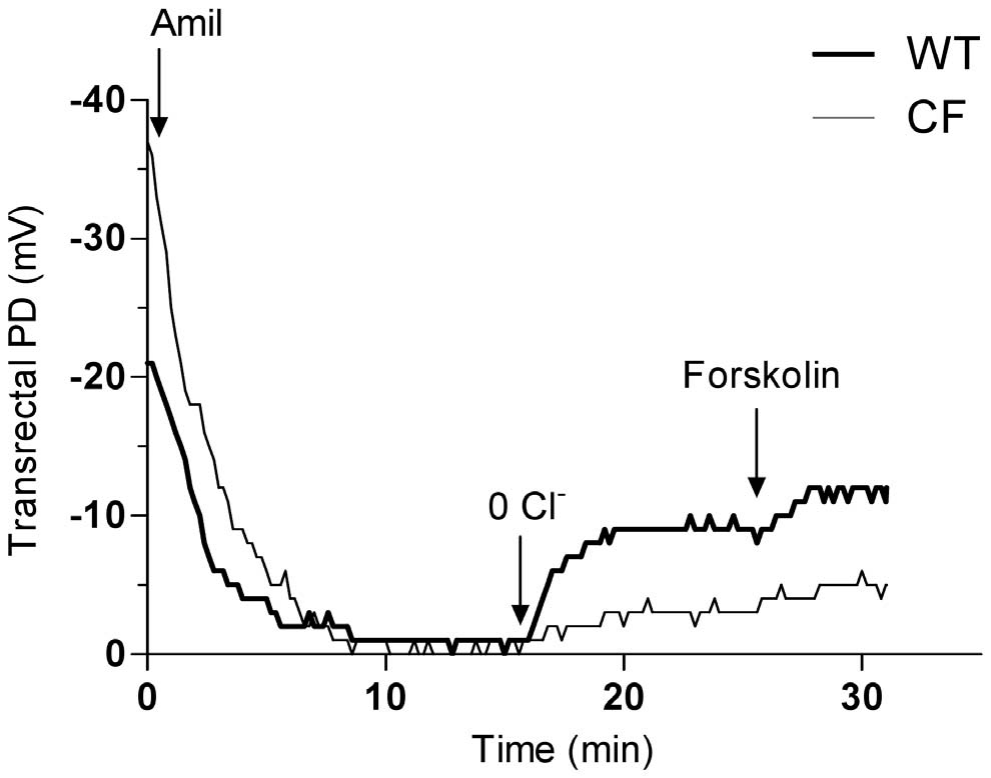
Representative tracings of transrectal potential difference (PD) measurements in baseline conditions in a wild-type mouse and a F508del homozygous mouse. Tracings show sequential response of the rectal mucosa to perfusion successively with Ringer solution, Ringer solution containing barium and amiloride (Amil), chloride-free solution containing barium and amiloride (0 Cl^−^), and chloride-free solution with barium, amiloride and forskolin. Arrows indicate time of solution changes.

**Figure 2:**
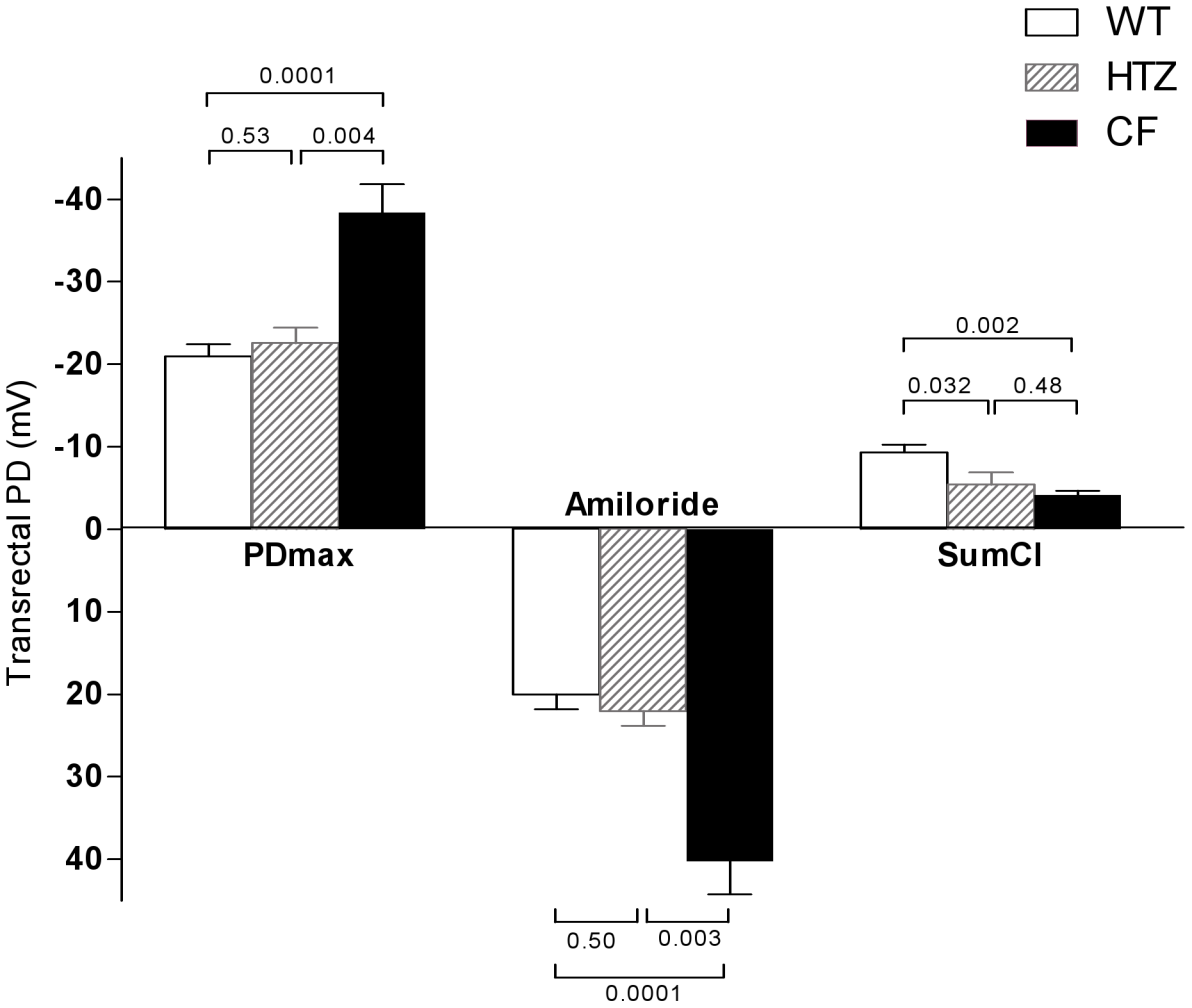
Maximal transrectal PD values (PDmax), response to amiloride and chloride transport (SumCl) in saline-treated wild-type (WT), heterozygous (HTZ) and homozygous (CF) mice for F508del mutation. Chloride transport was evaluated by the cumulative changes in transrectal PD after perfusion with chloride-free solution in the presence of barium, amiloride plus forskolin. Data are presented as means (±SEM) for 11, 5 and 5 animals in the wild-type and in the F508del heterozygous and homozygous groups respectively. P values denote levels of significance of between-group comparisons for the same transrectal PD parameter.

### Degree of Integrity of CFTR Function across the Intestinal Mucosa of Heterozygotes

To test the degree of integrity of intestinal CFTR function in heterozygotes, transrectal PD was measured in mice heterozygous for F508del-CFTR mutation and the values were compared with those obtained in normal homozygous and in F508del homozygous mice from the same genetic background. As illustrated in Figure 2, sodium transport, evaluated by the maximal stable basal voltage or by the response to amiloride, was preserved but chloride transport was lower in heterozygotes compared to normal homozygotes. These data indicate that mice heterozygous for the F508del-CFTR mutation have less functional intestinal CFTR with a reduced ability to transport chloride.

### Effect of Vardenafil on Transrectal PD Values in F508del Homozygous and Heterozygous Mice and in Wild-type Mice

To test whether GI epithelium is a target of the CFTR activating effect of therapeutic doses of PDE5 inhibitors [34], [35], we performed transrectal PD in F508del homozygous and heterozygous mice and in wild-type mice 1 hour after a single intraperitoneal injection of 50 µl of 0.07 mg/ml vardenafil dissolved in saline. The final administered dose of 0.14 mg/kg body weight was chosen in order to correspond to a human therapeutic dose used to treat erectile dysfunction (10 mg vardenafil for a 70-kg man). The same volume of 50 µl/25 g body weight of saline solution was injected in control experiments. Treatment with the PDE5 inhibitor was well tolerated and no adverse effect was observed.

Vardenafil did not induce any noticeable effect on sodium transport in either wild-type, F508del heterozygous or homozygous mice. However, a significant effect on chloride transport was detected, particularly in the presence of the F508del-CFTR mutation. Representative tracings obtained after vardenafil administration in the three groups of mice are shown in Figure S1A–C, and mean transrectal PD values are given in Figure 3. In the wild-type group, no significant increase of chloride transport was observed after treatment with vardenafil. The effect of vardenafil was at least twice as large in the F508del heterozygous and homozygous groups as in the corresponding saline-treated groups. In the heterozygous group, values were even larger than those obtained in the saline-treated wild-type group (see Table S1 for mean data). These data indicate that vardenafil is able, in the presence of the F508del-CFTR protein, either in the homozygous or the heterozygous status, to increase chloride transport across the GI epithelium without affecting sodium transport.

**Figure 3:**
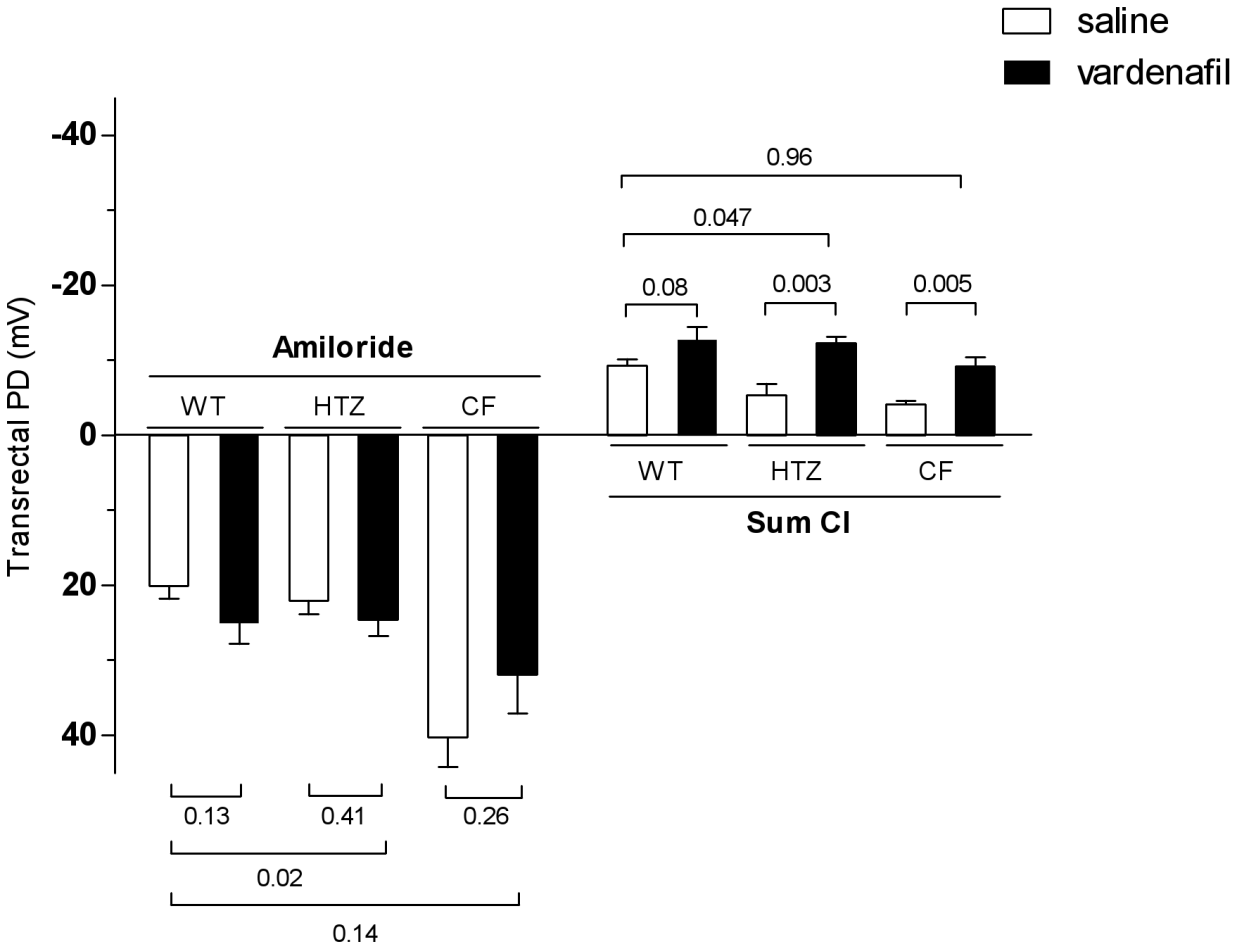
Influence of treatment with a single intraperitoneal dose of 0.14/kg vardenafil (vard) or saline on sodium and chloride transport in wild-type (WT), heterozygous (HTZ) and homozygous (CF) mice for the F508del mutation. Sodium transport evaluated by response to amiloride. Chloride transport evaluated by the cumulative changes in transrectal PD after perfusion with chloride-free solution in the presence of barium, amiloride plus forskolin. Data are shown as means (± SEM) for 5–11 animals per group. P values denote levels of significance of between-group comparisons for the same component of the chloride transport.

### Influence of Vardenafil on the Separate Components of Chloride Transport

We next analyzed the influence of the treatment with vardenafil on the relative contributions of the components of the chloride transport, namely the chloride gradient-dependent and the forskolin-dependent fractions. In the absence of vardenafil treatment, the chloride gradient-dependent component represents the major (4/5) fraction of the global chloride transport in the wild-type group (Figure 4). In the presence of the F508del-CFTR mutation, the chloride gradient-dependent fraction was similarly reduced in the homozygous as in the heterozygous group. However, the response to forskolin, virtually lost in the homozygous group, was preserved in the heterozygous group. Treatment with vardenafil influenced both fractions with distinct effects depending on the genotype. In all groups, the effect of the PDE5 inhibitor on the forskolin component was relatively larger than that on the chloride gradient-dependent fraction. In the heterozygous group, values reached after drug treatment were 4-fold larger than those recorded in the corresponding saline-treated group and the relative minor contribution of the forskolin-dependent fraction changed from about 1/5 (as seen in saline-treated wild-type mice) to almost a half of the global chloride transport. In the F508del homozygous group, the rescue of chloride transport by treatment with vardenafil resulted from the association of stimulating effects on both the chloride gradient-dependent and the forskolin-dependent fractions. Table S1 gives mean data.

**Figure 4:**
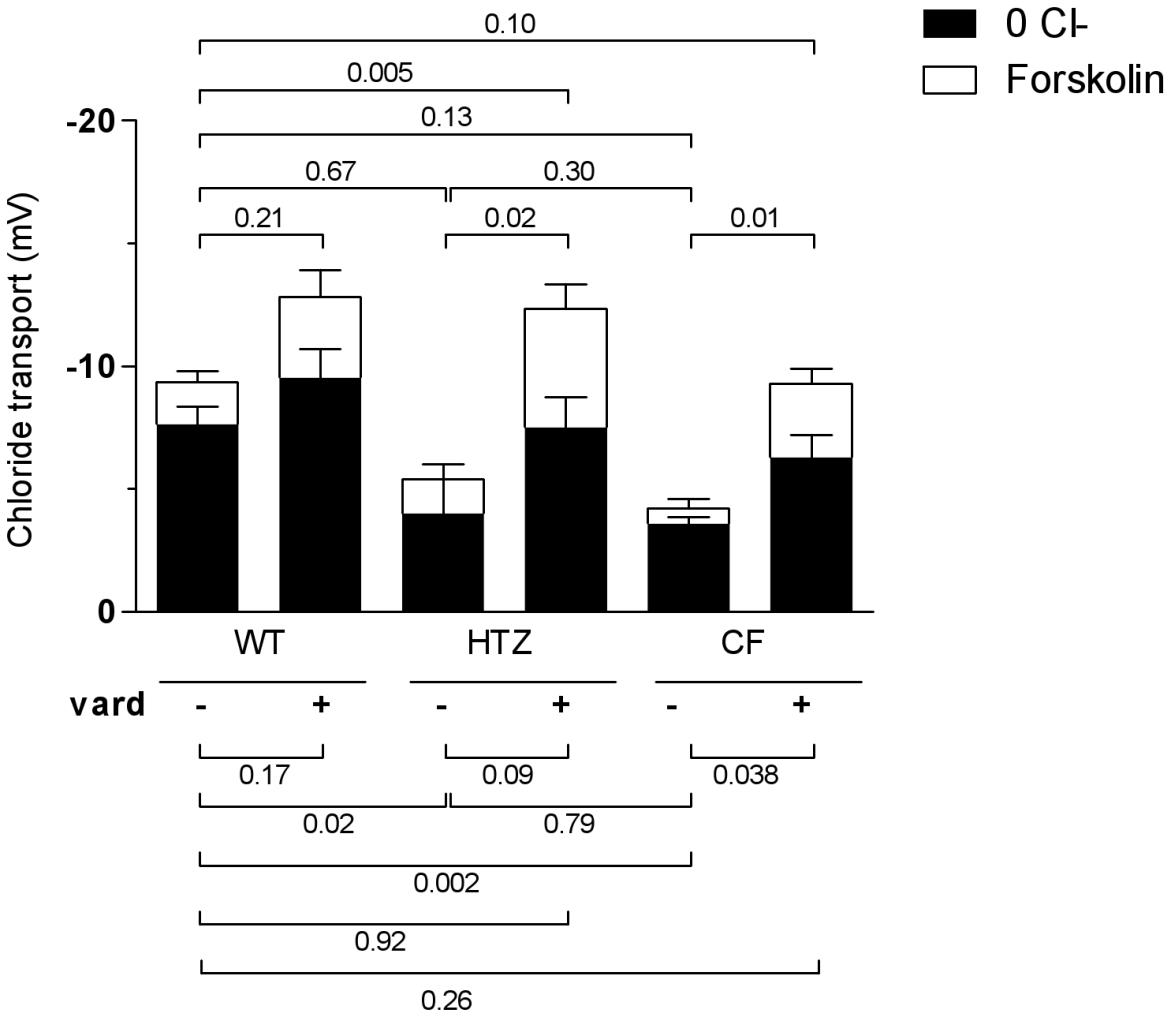
Influence of treatment with a single intraperitoneal dose of 0.14/kg vardenafil (vard) on the separate components of chloride transport in wild-type mice (WT), in mice heterozygous (HTZ) and homozygous (CF) for the F508del mutation. Responses of the rectal mucosa to perfusion with chloride-free solution containing barium and amiloride and responses of the rectal mucosa to the further addition of forskolin are shown as means (± SEM) for 5–11 animals per group. P values denote levels of significance of between-group comparisons for the same component of the chloride transport.

These data show that the transrectal PD test allows dissecting GI transepithelial ion transport properties and that vardenafil potentiates cAMP-mediated chloride transport in the presence of the F508del-CFTR or the wild-type protein. The data also indicate that the reduced ability to transport chloride in heterozygous status is associated with a preserved cAMP mediation of chloride transport activity.

### Immunohistochemical Expression and Localization of CFTR Protein in Mouse Colon Preparations

To substantiate transrectal PD data, we performed immunohistochemical localization studies of endogenously expressed CFTR on native colon tissues from 129/FVB F508del homozygous and wild-type mice 1 hour after an intraperitoneal injection of saline. Permeabilized mouse distal colon cryosections were stained for CFTR using a monoclonal anti-CFTR antibody raised against the intracellular C-terminus (clone 24-1) recognizing both the wild-type and the F508del protein [39]. Representative images of colon cryosections showing the CFTR signal, revealed with Alexa Fluor 488 conjugated antibodies and detected by fluorescence microscopy, are illustrated in Figure 5A–F. Specificity of the CFTR signal was verified in cryosections from colon of *Cftr* knockout mice (Figure 5A) and by the absence of signal when no primary antibody was used (Figure 5B). In colon sections from wild-type mice, the immunofluorescence CFTR signal was mostly detected at the apical cell compartment of colonocytes from intestinal crypts (Figure 5C) while a reduced signal was obtained in saline-treated F508del-CF colon tissues (Figure 5D). This finding indicates that the mutant protein is mislocalized with a reduced expression in the plasma membrane compartment.

**Figure 5:**
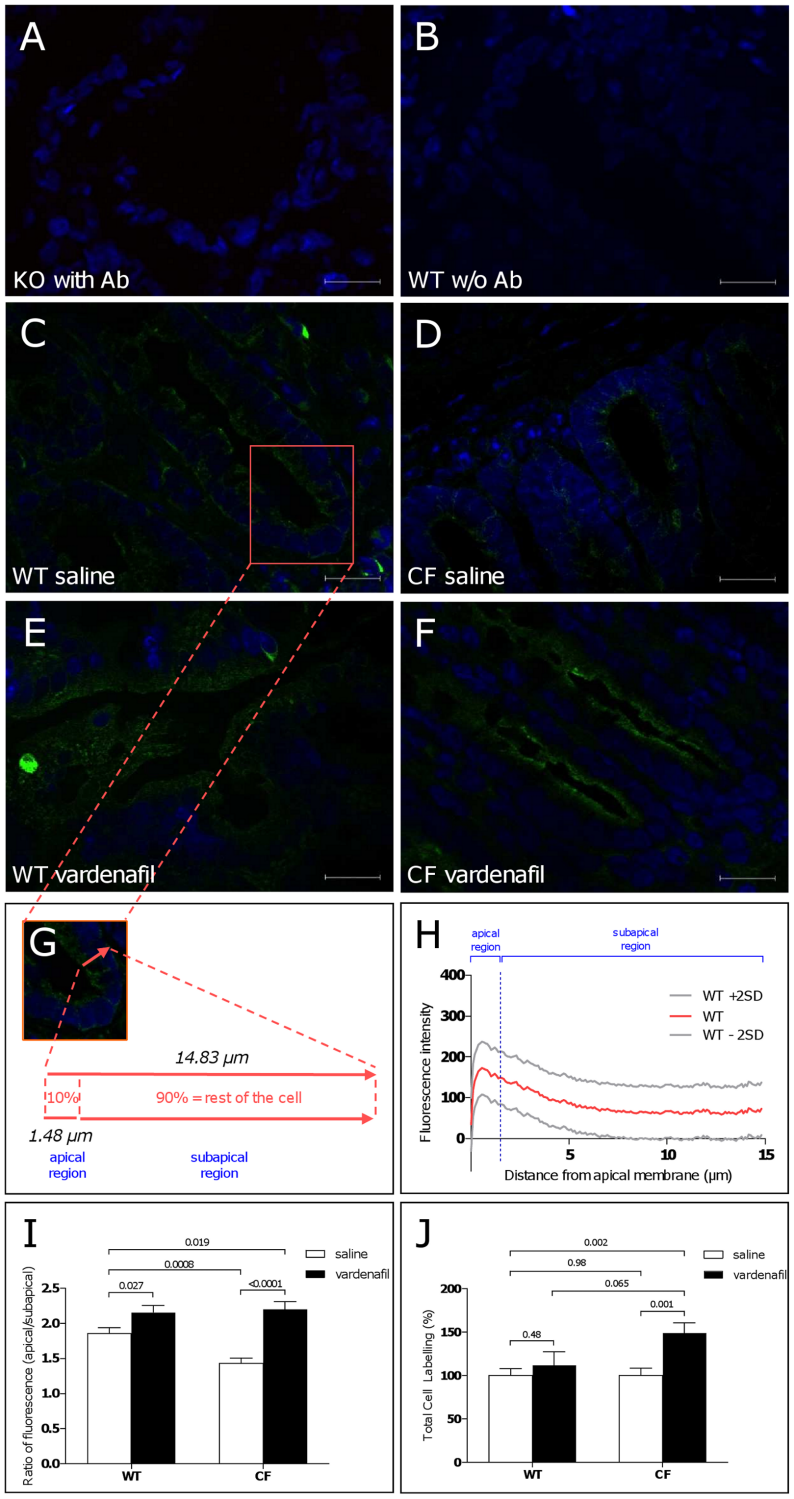
Immunohistochemical localization studies showing absence of labelling of endogenously expressed CFTR in distal colon tissue from a Cftr knockout mouse (A) and a wild-type mouse (B) in the absence of primary anti-CFTR antibody (w/o Ab). Immunolabelling performed 1 hour after a single intraperitoneal injection of saline (C,D) or 0.14 mg/kg vardenafil (E,F) in crypt colonocytes from a wild-type mouse (C,E) or a F508del-CF homozygous mouse (D,F). Vardenafil treatment (E,F) increased CFTR (green) labelling at the apical membrane compartment. Nuclei (blue labelling) stained by DAPI. Morphometric analysis of crypt cells with measure of the apical and subapical (corresponding to the rest of the cell height) compartments (G). Mean values and upper/lower 95% confidence intervals (±2SD) of scans of the intensity of the CFTR fluorescence signal along a line drawn through the apical to the basal cell borders obtained from 136 crypt colonocytes from saline-treated wild-type mice; the vertical line marks the apical compartment corresponding to the upper 10% of the height of the cell; total area under the curve = 1285 µm.intensity unit; area under the curve of the apical region = 219.6 µm.intensity unit; peak intensity = 172.8 units; distance from apical membrane to peak intensity = 0.555 µm (H). Normalized ratio of apical/subapical fluorescence CFTR signal (I) and total cell labelling (J) in saline-treated and vardenafil-treated mice.

### Influence of Vardenafil on CFTR Protein Localization and Distribution in Mouse Colonocytes

To better assess the potential mechanism(s) involved in the activating effect of vardenafil on cAMP-dependent chloride transport across the rectal mucosa, we next examined the cellular expression and distribution of CFTR protein after treatment with the PDE5 inhibitor (1 hour after a single 0.14 mg/kg dose as used in transrectal PD experiments). CFTR expression at the apical region of crypt colonocytes from F508del-CF mice was increased by vardenafil (compare Figures 5D and 5F). This data was confirmed by morphometric analysis of randomly assigned crypt colonocytes and by scanning the fluorescence intensity of the CFTR signal along a line drawn through the apical to the basal cell borders, corresponding to the total height of cells (Figure 5G). The distribution of the fluorescence CFTR signal at both apical (corresponding to the upper 10% of the cell height) and subapical (corresponding to the rest of the cell height) compartments was quantified morphometrically in samples from 4 animals per group and 26–38 cells per mouse (*i.e*. n = 104–152 cells per group). As shown in Figure 5H, the fluorescence signal of wild-type CFTR was mostly targeted to the apical cell compartment where a peak of intensity was found, indicating that the wild-type protein is localized at the apical region of cells. As illustrated in Figure S2A–C, the reduced area under the fluorescence curve (175.4 µm.intensity) of the apical compartment of colonocytes from F508del-CF mice was largely increased after treatment with vardenafil (323.2 µm.intensity). These data indicate that vardenafil promotes a redistribution of the CFTR protein among cell compartments towards the apical region. The peak of the fluorescence intensity scan was located beneath the apical region in colonocytes from F508del-CF mice, revealing that the mutant protein accumulates in subapical regions and does not reach the apical membrane (Figure S2A).

To further analyze the redistribution of CFTR protein, a normalized ratio of the apical/subapical fluorescence CFTR signal was calculated. A 25% loss of the ratio was found in colonocytes from F508del-CF mice compared to those obtained from wild-type animals (Figure 5I), while no difference was found in total cell labeling (Figure 5J). Vardenafil treatment increased the ratio in tissue preparations from F508del-CF mice (Figure 5I). In wild-type mice, vardenafil also increased both the apical/subapical fluorescence ratio (Figure 5I) and the peak of intensity of the CFTR signal without changing its location within the apical compartment (Figure 5H). Means ± upper/lower confidence interval of individual fluorescence intensity scans obtained from crypt colonocytes from vardenafil-treated wild-type and F508del-CF mice are shown in Figure S2B,C. Vardenafil did not affect the total cellular CFTR labeling in the wild-type group as it did in the F508del-CF group (Figure 5J). Altogether, these data show that the F508del-CFTR protein spans mostly within a compartment beneath the apical membrane region of crypt colonocytes and that vardenafil promotes its accumulation and redistribution towards the transmembrane region.

## Discussion

The introduction of CF mouse models has marked a significant milestone in the efforts to further our understanding of CF pathophysiology and more recently to search for the efficacy of novel drugs for the treatment of CF. The F508del-CF mouse we used in this study mimics human CF disease in several aspects [36]. In particular, intestinal disease is the primary phenotype of the mouse model which presents with a meconium ileus-like disease requiring, from weaning, addition of an osmotic laxative to drinking water in order to prevent fatal intestinal obstruction [35].

The present work was designed to test the hypothesis that the cGMP-specific PDE5 inhibitor vardenafil, administered *in vivo* at clinical doses, rescues the loss of chloride channel function and the mislocalization of F508del-CFTR in the GI tract predominantly affected in CF. Because the drug is in clinical use, preclinical studies using animal models of the human disease are of great relevance for characterizing its beneficial effects, mechanisms of action and target organs before moving towards a new clinical application.

Identifying a therapeutic strategy that combines ability to correct the basic ion transport defect at multitarget organs, to exert an anti-inflammatory effect [40] and to control deregulated proinflammatory and fibrogenic phenotype of CF fibroblasts [41], is very exciting and promising. Indeed, lung inflammation and tissue remodeling and fibrosis contribute to the pathogenesis of CF and are influenced by vardenafil [40], [41]. Results from ongoing phase 1/2 studies aimed at testing the effect of sildenafil on CFTR-dependent ion transport activity through nasal PD measurements and on lung inflammation (listed on clinicaltrials.gov, NCT 01132482 and 00659529) are awaited.

The effects of therapeutic strategies aimed at correcting the CF electrophysiological phenotype in affected epithelia has also been clinically assessed *ex vivo* by examining rectal biopsy specimens mounted in Ussing chambers [42]. Similarly, a reliable *in vivo* assay of CFTR function in intestinal epithelia of preclinical CF mouse models is extremely valuable to study efficacy of pharmacological interventions. Our data point to the rectal mucosa as an additional target tissue to study *in vivo* basic ion transport defects in CF mice. The transrectal PD test is reliable and has been previously validated [43]. It allows discriminating between CF and non-CF animals and dissecting transepithelial ion conductances and responses to pharmacological and non-pharmacological stimuli. Moreover, the test is little invasive and is followed by full recovery, allowing repeated serial assessments in the same animal. As shown for the CF mouse nasal PD [34], [35], [37], [38], the transrectal PD allows a clear-cut *in vivo* discrimination between CF and wild-type mice, with decreased chloride transport with near-null cAMP-stimulated response reflecting loss of function of CFTR and increased sodium transport reflecting overfunctional ENaC. Interestingly, mice heterozygous for the F508del mutation present reduced functional chloride transport but preserved sodium transport. One wild-type CFTR allele appears to be sufficient to ensure integrity of sodium transport while two alleles are required to ensure integrity of chloride transport. Our data support the heterozygote selective advantage theory assuming that a selective advantage of resistance to cholera is a possible explanation for the high frequency of CF mutations in the Caucasian populations. It has been postulated that CFTR protein mediates toxin-induced secretory diarrhoea and that heterozygotes, having a less functional CFTR, were protected from dehydration due to diarrheal diseases caused by toxins of *Vibrio cholera* and *Escherichia coli*. Our data are in line with the findings that CF heterozygous mice have half the normal intestinal fluid efflux after exposure to cholera toxin [44] and that intestine of patients with CF does not actively secrete chloride in response to a variety of secretagogues [45].

The activating effect of vardenafil on fractional components of chloride transport we have observed in the rectal mucosa of mice parallels what we have previously reported for the nasal mucosa [34], [35]. The fact that the response to forskolin was largely influenced by vardenafil treatment, even in the presence of wild-type CFTR, suggests intimate cross-talk between the cAMP and cGMP signal transduction pathways in the modulation of CFTR channel activity and supports the view that the drug acts as a CFTR channel gating potentiator. In submandibular acinar cells expressing a CF-like phenotype, the corrective effect of PDE inhibitors on CFTR-mediated mucin defects was shown to involve increased cellular levels of cGMP [46]. It has been postulated that intracellular accumulation of cGMP inhibits the action of PDE3, responsible for the degradation of cAMP [46]. By contrast, the fact that rises in cAMP concentration produced by cAMP-specific PDE inhibitors do not parallel the resulting increases in chloride transport across Calu-3 cells [47] is in keeping with the assumption that PDE inhibitors may affect CFTR through cAMP-independent mechanisms [48]. As in the nasal mucosa, the lack of effect of vardenafil on electrogenic sodium transport might argue against a direct reciprocal relationship between CFTR and ENaC activity in mouse native tissues.

The intestinal distribution of CFTR in rodents resembles that of human [49]. Cellular distribution studied by immunohistochemistry staining of colon native tissues confirmed that wild-type CFTR protein is mainly located in the region of the apical membrane of crypt colonocytes, which are the sites of intestinal fluid and electrolyte secretion [31]. Quantification of the cellular distribution of CFTR in crypt colonocytes supported the notion that the F508del-CFTR protein accumulates in a subapical vesicular compartment beneath the luminal membrane and that the mutant protein fails to escape from the ER to be delivered to the plasma membrane.

The effect of vardenafil on redistribution of the mutant and of the wild-type CFTR protein from the subapical to the apical compartment in crypt colonocytes indicates that the drug acts as a CFTR corrector. Vardenafil may act by favoring protein glycosylation and by correcting organellar hyperacidification in CF cells. Indeed, it has been shown that sildenafil normalizes luminal pH in the trans Golgi network of CF epithelial cells [50]. Yet another possibility, based on *in vitro* studies, would be that vardenafil influences phosphorylation of the R domain of CFTR by PKG to then modify PKA-mediated phosphorylation [30]. In rat jejunum, cGMP induced a large increase in surface CFTR in enterocytes in association with fluid secretion that was inhibited by PKG inhibitors [51]. It has been concluded that cAMP and cGMP-dependent phosphorylation regulates fluid secretion and CFTR trafficking to the surface of enterocytes in rat jejunum [51].

Consistent with published data [52], the PDE5 inhibitor acts both as a corrector and as a potentiator.

Finally, our findings point at the intestinal mucosa as a valuable target tissue to study CFTR function and localization and to evaluate efficacy of therapeutic strategies in CF. By using two independent techniques, we showed that, as in airways, therapeutic doses of vardenafil are able to target in the GI tract, predominantly affected in CF, multiple molecular defects caused by the F508del-CFTR mutation. Acting as a CFTR potentiator, the drug exerts a short-term activation of transepithelial cAMP-dependent chloride transport not only in the F508del homozygous status but also in the presence of wild-type CFTR or of F508del heterozygous status. Acting as a CFTR corrector, it promotes acute accumulation and redistribution of the protein towards and into the apical compartment where the wild-type protein is mainly expressed. The study provides compelling support for targeting the cGMP signaling pathway in CF pharmacotherapy.

## Materials and Methods

### Animal Model

Young adult (12–16 weeks old, 20–30 g) 129/FVB *Cftr^tmi1EUR^* mice homozygous for the F508del-CFTR mutation [36] were housed under conventional conditions. C57BL/6 *Cftr* ^UNC^ knockout mice were also investigated in immunohistostaining analyses. The genotype of each animal was checked at 21 days of age using Taqman quantitative PCR as previously described [53]. Experiments were approved by the local Ethics Committee for animal research at the Université catholique de Louvain (2010/UCL/MD/034) and conformed to the European Community regulations (CEE n° 86/609).

### Vardenafil Treatment

Stock solutions of 0.07 mg/ml vardenafil HCl (Bayer, West Haven, Germany) prepared in saline were stored at 4°C and used within 4 days after preparation. Vardenafil (0.14 mg/kg body weight) was applied as a single intraperitoneal dose. The same volume of sterile saline was injected in control experiments. Experiments were performed 1 h after the injection.

### Transrectal PD Measurements

Transrectal PD measurements were adapted from the nasal PD technique [34], [35], [37], [38]. Anaesthetized mice were lying on their backs on a heating pad, and paws and tail were taped out of the way. A ∼0.2 mm double-lumen catheter was inserted 0.5 cm into the rectum, one lumen being used to perfuse Ringer solutions; the other one served as a measuring Ag/AgCl electrode (SLE Instruments, South Croydon, UK) and was connected to the positive terminal of a data memory high-impedance (>10^12^ Ω) voltmeter (Knick Portamess 913; Electronishe Mebgeräte, Berlin, Germany) through an electrode cream (Signa cream; Parker Labs, Fairfield, NJ) diluted 1∶1 (vol/vol) in 3 M KCl. An intravenous catheter filled with the similarly diluted electrode cream was inserted subcutaneously and served as a bridge for connecting the reference electrode. Solutions were perfused at a constant rate of 12 µl/min in the following sequence: 1) Ringer solution (140 mM Na^+^, 120 mM Cl^−^, 5.2 mM K^+^, 25 mM HCO_3_^−^, 2.4 mM HPO_4_^−^, 0.4 mM H_2_PO_4_^−^, 1.2 mM Ca^2+^, 1.2 mM Mg^2+^; pH 7.4); 2) Ringer solution with 0.1 mM amiloride and 5 mM barium hydroxide; 3) chloride-free Ringer solution with amiloride/barium, and 4) amiloride/barium chloride-free Ringer solution with 10 µM forskolin. In the chloride-free Ringer solutions, chloride was replaced by osmotically equivalent gluconate.

### Immunohistochemistry

Freshly excised mouse distal colon specimens were washed in PBS solution and placed in a cryomold (Sakura, Tissue-Tek, Torrance, CA) embedded in OCT compound and frozen in liquid nitrogen vapors. Cryosections (7 µm) were fixed in acetone for 10 min at 4°C [54]. Tissues slides were incubated in 0.25% Triton X-100 in PBS to permeabilize the membranes. Sections wiped with a histology Dako Pen (S2002, Glostrup, Denmark) were incubated for 1 h in blocking reagent M.O.M basic kit (Vector Labs, Peterborough, UK) following manufacturer’s instructions. Sections were incubated overnight at 4°C with primary anti-CFTR monoclonal antibody raised against the C-terminus (clone 24-1, MAB25031, R&D Systems, UK) diluted 1∶100 [55]. Negative controls omitting primary antibodies were prepared in parallel. After rinsing three times in 0.1% Triton X100 in PBS, slides were incubated for 1 h at room temperature with goat anti-mouse secondary antibody (Alexa Fluor 488 IgG (H+L), 2 mg/ml, Invitrogen, Belgium) diluted 1∶1000 in 0.1% Triton X-100 in PBS for CFTR staining. Slides were washed in PBS and mounted in Vectashield anti-fading medium containing DAPI (1.5 µg/ml, Abcys, France) for nuclear labelling. Labelled sections covered with a cover slide and sealed with nail polish were stored at 4°C in the dark. Tissue sections were imaged by structured illumination microscopy using a Zeiss AxioImager Z1 fluorescent microscope equipped with an ApoTome module. Images taken with an exposition time of 40 ms were exported to AxioVision Release 4.8.2.0 for quantification analyses. Morphometric analyses were performed using larger magnification images (63×; numerical aperture 1.4; oil immersion).

### Statistics

Descriptive statistics (mean ± SEM) and tests of statistical significance were performed using GraphPad Prism 5 (GraphPad Software Inc, La Jolla, CA, USA). Prior to statistical analysis, the data were checked for normality of distributions (Shapiro-Wilk normality test). Between-group comparisons were evaluated using one-way ANOVA test. Posthoc comparisons were made using Student’s *t* test or Tukey-Kramer HSD test, for two or more than two x levels. Peak of the fluorescence intensity scan and area under the curve were calculated by nonlinear regression analyses using GraphPad Prism5. Null hypothesis was rejected at p-values <0.05.

## Supporting Information

Figure S1 **Representative tracings of transrectal potential difference (PD) measurements obtained in vardenafil-treated and saline-treated wild-type mouse (A), F508del heterozygous mouse (B) and F508del homozygous mouse (C).** Tracings show sequential response of the rectal mucosa to perfusion successively with Ringer solution, Ringer solution containing barium and amiloride (Amil), chloride-free solution containing barium and amiloride (0 Cl^−^), and chloride-free solution with barium, amiloride and forskolin. Arrows indicate time of solution changes.
(TIF)

Figure S2 **Mean values and upper/lower 95% confidence intervals (±2SD) of scans of the intensity of the CFTR fluorescence signal along a line drawn through the apical to the basal cell borders obtained from 152 crypt colonocytes from saline-treated F508del-CF mice (A); from 104 crypt colonocytes from vardenafil-treated wild-type mice (B) and from 128 crypt colonocytes from vardenafil-treated F508del-CF mice (C).** In colonocytes from saline-treated F508del-CF mice (panel A): total area under the curve = 1376 µm.intensity unit; area under the curve of the apical region = 175.4 µm.intensity unit; peak intensity = 158.4 units; distance from apical cell membrane to peak intensity = 1.48 µm; total cell height = 13.42 µm. In colonocytes from vardenafil-treated wild-type mice (panel B): total area under the curve = 1248 µm.intensity unit; area under the curve of the apical region = 228.9 µm.intensity unit; peak intensity = 211.3 units; distance from apical membrane to peak intensity = 0.741 µm; total cell height = 13.7 µm. In colonocytes from vardenafil-treated F508del-CF mice (panel C): total area under the curve = 1742 µm.intensity unit; area under the curve of apical region = 323.2 µm.intensity unit; peak intensity = 284 units; distance from apical membrane to peak intensity = 0.555 µm; total cell height = 14.07 µm. Vertical lines mark the apical compartment corresponding to the upper 10% of the height of the cell.
(TIF)

Table S1 **Influence of treatment with a single intraperitoneal dose of 0.14 mg/kg vardenafil or saline on total and on separate components of chloride transport, i.e. the chloride gradient-dependent and the forskolin-dependent fractions in wild-type (WT) mice and in mice heterozygous (HTZ) and homozygous (CF) for the F508del-CFTR mutation.** Data are means (± SEM) for 5–11 animals per group.
(XLS)
