# Supplementary figures and images for: Correction of Chloride Transport and Mislocalization of CFTR Protein by Vardenafil in the Gastrointestinal Tract of Cystic Fibrosis Mice
Source: PLoS One. 2013 Oct 24;8(10):e77314. doi: 10.1371/journal.pone.0077314 (PMC3811977; doi:10.1371/journal.pone.0077314)

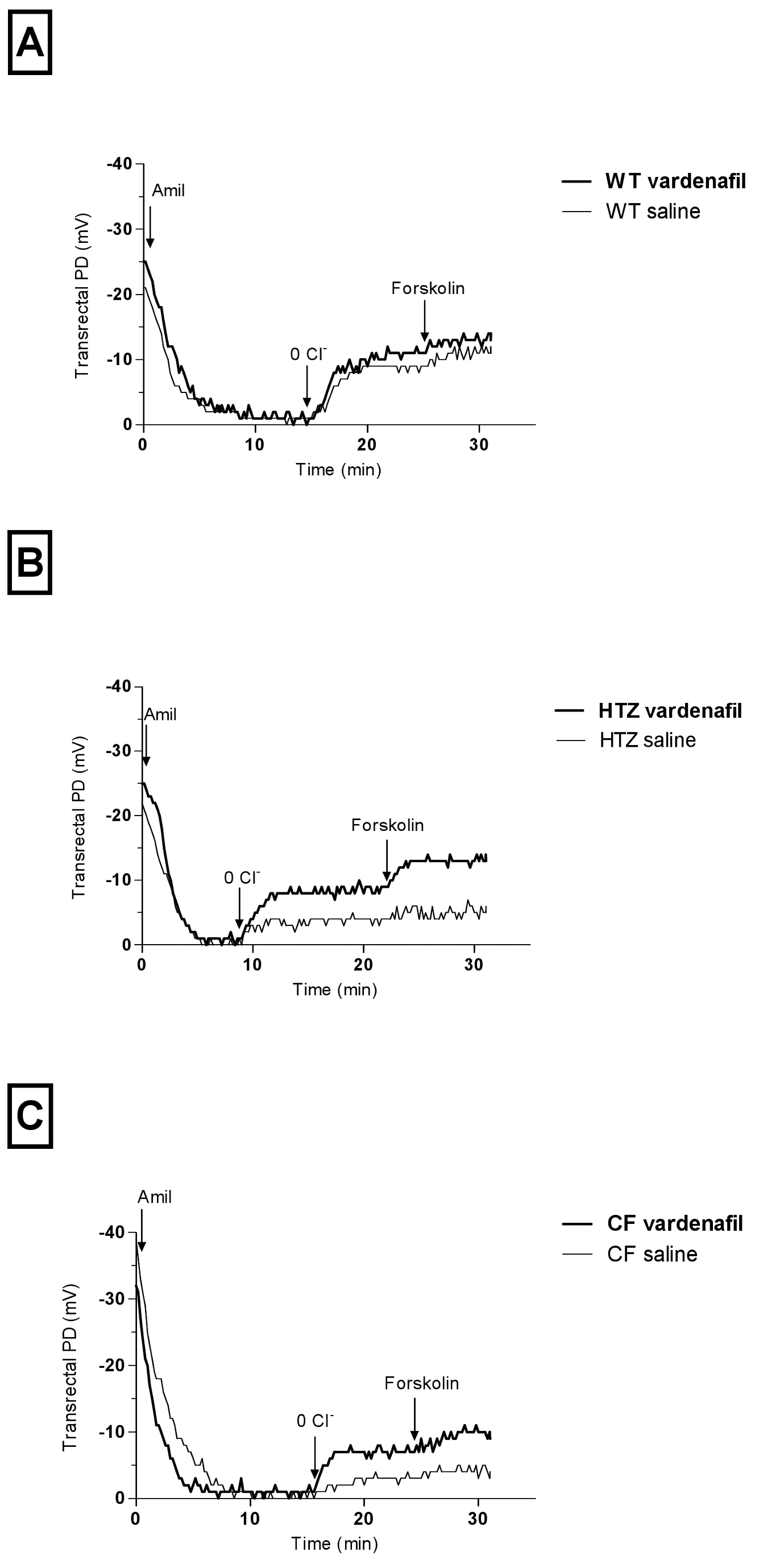

Supplement: Figure S1 — Representative tracings of transrectal potential difference (PD) measurements obtained in vardenafil-treated and saline-treated wild-type mouse (A), F508del heterozygous mouse (B) and F508del homozygous mouse (C). Tracings show sequential response of the rectal mucosa to perfusion successively with Ringer solution, Ringer solution containing barium and amiloride (Amil), chloride-free solution containing barium and amiloride (0 Cl−), and chloride-free solution with barium, amiloride and forskolin. Arrows indicate time of solution changes. (TIF) [file pone.0077314.s001.tif]

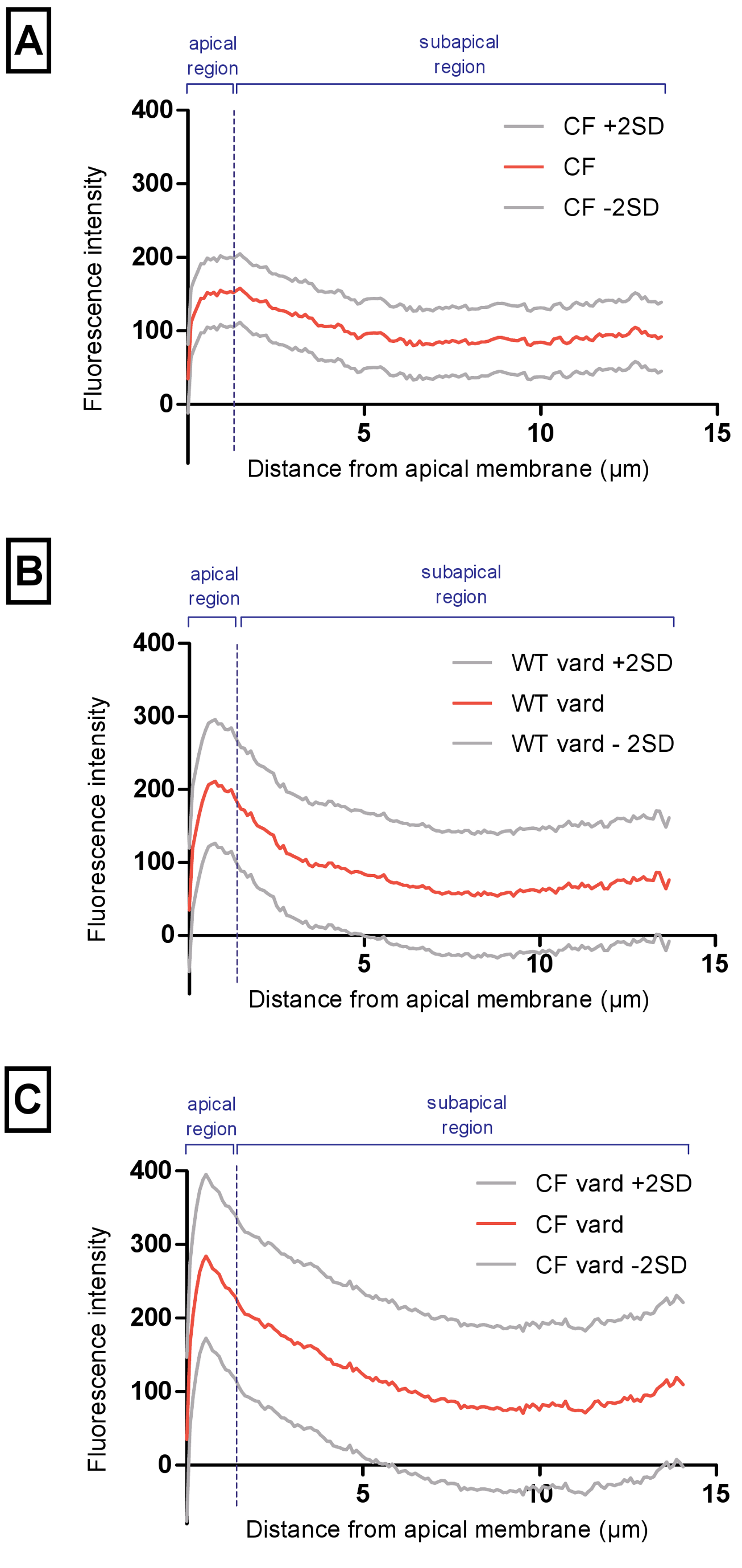

Supplement: Figure S2 — Mean values and upper/lower 95% confidence intervals (±2SD) of scans of the intensity of the CFTR fluorescence signal along a line drawn through the apical to the basal cell borders obtained from 152 crypt colonocytes from saline-treated F508del-CF mice (A); from 104 crypt colonocytes from vardenafil-treated wild-type mice (B) and from 128 crypt colonocytes from vardenafil-treated F508del-CF mice (C). In colonocytes from saline-treated F508del-CF mice (panel A): total area under the curve = 1376 µm.intensity unit; area under the curve of the apical region = 175.4 µm.intensity unit; peak intensity = 158.4 units; distance from apical cell membrane to peak intensity = 1.48 µm; total cell height = 13.42 µm. In colonocytes from vardenafil-treated wild-type mice (panel B): total area under the curve = 1248 µm.intensity unit; area under the curve of the apical region = 228.9 µm.intensity unit; peak intensity = 211.3 units; distance from apical membrane to peak intensity = 0.741 µm; total cell height = 13.7 µm. In colonocytes from vardenafil-treated F508del-CF mice (panel C): total area under the curve = 1742 µm.intensity unit; area under the curve of apical region = 323.2 µm.intensity unit; peak intensity = 284 units; distance from apical membrane to peak intensity = 0.555 µm; total cell height = 14.07 µm. Vertical lines mark the apical compartment corresponding to the upper 10% of the height of the cell. (TIF) [file pone.0077314.s002.tif]
